# A rare midbrain infarction presenting with plus-minus lid syndrome with ataxia: a case report

**DOI:** 10.1186/1752-1947-5-525

**Published:** 2011-10-25

**Authors:** Khalid Alsherbini, Kevin Kapadia, Justin A Sattin

**Affiliations:** 1 Neurology Department, University of Wisconsin - Madison, 1685 Highland Avenue, Medical Foundation Centennial Building, Madison, WI 53705-2281, USA

## Abstract

**Introduction:**

We present the case of a patient with midbrain infarction with an unusual clinical presentation, where clinical diagnosis and anatomical localization were valuable tools in deciding treatment.

**Case presentation:**

Our patient was a 59-year-old, right-handed Caucasian man with hypertension who presented to our facility with acute diplopia that persisted until he developed complete right-sided ptosis. He also had difficulty walking and coordinating movements of his upper extremities bilaterally, but this was worse on his left side.

**Conclusions:**

Plus-minus lid syndrome with ataxia is a rare presentation of midbrain infarction with a unique localization and anatomical description. This case highlights the importance of clinical skills for making a diagnosis in the absence of imaging to confirm the findings.

## Introduction

A total of 20% of ischemic strokes involve structures supplied by the posterior (vertebrobasilar) circulation. Many cases remain undiagnosed or incorrectly diagnosed because of the atypical clinical presentation and the lower sensitivity of diagnostic imaging of the posterior fossa. Although lacunar infarctions are usually less than 15 mm in diameter, the symptoms might be clinically devastating, especially when the midbrain or pons is affected. Such lesions often are very challenging to localize and require a thorough knowledge of neuroanatomy.

## Case presentation

Our patient was a 59-year-old, right-handed Caucasian man with hypertension who presented to our facility with acute diplopia that persisted until he developed complete right-sided ptosis. He also had difficulty walking and coordinating movements of his upper extremities bilaterally, but this was worse on his left side.

**Figure 1:**
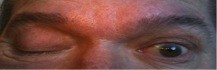
**Our patient's right eye showing complete ptosis with left lid retraction**.

**Figure 2:**
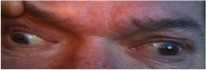
**Our patient's right eye deviated down and out with no correction of the left eye retraction with elevation of the right eyelid**.

**Figure 3:**
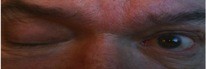
**Impairment of left eye upgaze due to superior rectus weakness; our patient was asked to look up and to the left**.

**Figure 4:**
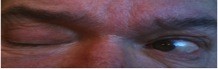
**Intact medial rectus muscle on the left**.

Physical examination showed a complete, pupil-sparing, right oculomotor nerve palsy (Figure 1 and Figure 2). He had left eyelid retraction that did not correct upon passively elevating the right eyelid, indicating that the retraction was not due to increased levator palpebrae superioris activity from attempted fixation with the ptotic eye (Figure 2). There was impaired upward gaze in the left eye due to superior rectus weakness (Figure 3) with intact medial rectus (Figure 4). He had no fatigable weakness and no sign of inflammation of the eye or surrounding soft tissues; a magnetic resonance imaging (MRI) scan of his brain showed no restricted diffusion.

On follow-up six months after an acute rehabilitation stay, his ptosis had resolved but he continued to have diplopia due to residual oculomotor paresis. His ataxia had improved with physical therapy such that he was able to walk with a cane inside his house and with a walker for longer distances.

## Discussion

The finding of ptosis on one side and eyelid retraction on the other is known as plus-minus lid syndrome, and was originally described by Gaymard *et al. *[1]. It is usually caused by injury to the third nerve fascicle and the nucleus of the posterior commissure, although it has also been described in ocular myasthenia gravis [2].

Two muscles control the eyelid: one is the superior tarsal muscle (Müller's muscle), which is innervated by sympathetic fibers and is thus affected in Horner's syndrome. The other is the levator palpebrae superioris, a skeletal muscle innervated by oculomotor fibers originating from the central caudal subnucleus of the third nerve complex [3,4].

The nucleus of the posterior commissure sends inhibitory fibers to the central caudal nucleus. Lesions of this structure result in disinhibition of levator palpebrae superioris bilaterally. When the oculomotor fascicle is also involved, as in our patient's case, the ipsilateral eyelid retraction is masked by the ptosis resulting from the infra-nuclear third nerve palsy [2-4].

The contralateral ataxia was attributed to involvement of the superior cerebellar peduncle (Claude syndrome). The contralateral superior rectus weakness improved quickly, but serves as a reminder of the unique contralateral innervation of this muscle [3,4].

An MRI of the brain had shown no restricted diffusion, which emphasizes the importance of making a clinical diagnosis. In one study, 25% of clinically diagnosed patients who had had a stroke had a negative initial diffusion-weighted MRI scan, with 23% of those having evidence of infarction on a repeat MRI scan. Most such lesions were lacunar and in the brainstem [5].

## Conclusions

Plus-minus lid syndrome with ataxia is a rare presentation of midbrain infarction with a unique localization and anatomical description. This particular case highlights the importance of clinical skills for making a diagnosis in the absence of imaging to confirm the findings.

## Consent

Written informed consent was obtained from the patient for publication of this case report and any accompanying images. A copy of the written consent is available for review by the Editor-in-Chief of this journal.

## Competing interests

The authors declare that they have no competing interests.

## Authors' contributions

KA analyzed and interpreted the initial data regarding our patient's presentation and performed the literature review, and wrote the initial manuscript. JS reviewed the initial draft and made corrections and final editing changes, and he added some neuro-anatomy review material. KK helped with the final editing and review of the manuscript, with finalizing of the images used. All three authors were the stroke care team for our patient. All authors read and approved the final manuscript.
